# Nomogram Based on Systemic Immune-Inflammation Index to Predict Overall Survival in Gastric Cancer Patients

**DOI:** 10.1155/2018/1787424

**Published:** 2018-12-02

**Authors:** Hongtai Shi, Youqin Jiang, Honggang Cao, Haiwen Zhu, Bin Chen, Weiwei Ji

**Affiliations:** ^1^Department of Radiotherapy, The Third People's Hospital of Yancheng, 75 Juchang Street, Yancheng 224005, China; ^2^Department of Medical Oncology, The Third People's Hospital of Yancheng, 75 Juchang Street, Yancheng 224005, China; ^3^Department of General Surgery, The Third People's Hospital of Yancheng, 75 Juchang Street, Yancheng 224005, China

## Abstract

**Background:**

The systemic immune-inflammation index (SII), based on peripheral lymphocytes, neutrophils, and platelet count, has been used as a prognostic marker for several tumors. However, use of the SII has not been reported for gastric cancer.

**Methods:**

We evaluated the prognostic value of the SII in primary and validation cohorts. We also established an effective prognostic nomogram for gastric cancer based on R language. The predictive accuracy and discriminative ability of the nomogram were determined using the concordance index (C index) and a calibration curve and were compared with TNM classifications.

**Results:**

The Kaplan-Meier survival analysis results showed that the high SII was associated with poor prognosis of gastric cancer patients in the primary and validation cohorts. SII proved to be related to tumor location, histological grade, tumor size, TNM stage, and perineural infiltration in patients with gastric cancer and was an independent prognostic factor for patients with gastric cancer. SII has a better predictive ability than other existing prognostic indexes based on inflammation, such as NLR, PLR, and MLR. The nomogram established can accurately predict the 3- and 5-year survival rates of patients with gastric cancer after operation, and its accuracy is significantly higher than that of the 8th edition of the AJCC staging system.

**Conclusion:**

SII can independently predict the overall survival of patients with gastric cancer after operation, which is superior to the existing systemic inflammatory indexes. The prognostic nomogram based on SII is a reliable model for predicting the postoperative survival of patients with gastric cancer.

## 1. Introduction

Gastric cancer is the fifth most common malignant tumor and the third leading cause of cancer death in the world and has become a major global health problem due to high morbidity and mortality [1, 2]. Although the incidence and mortality of gastric cancer have declined in the last decade, it is still the third most common cause of cancer death in Chinese men and the second most common cause of cancer in Chinese women [3]. Currently, surgery is still the main treatment for gastric cancer, but the survival rate of patients with gastric cancer is lower than 30% [4]. Recurrence or metastasis will occur in approximately 35–70% patients within 5 years, even after radical resection [5]. To reduce the risk of postoperative recurrence and metastasis, early diagnosis and developing an appropriate treatment plan based on the expected survival time of patients will help improve the cure rate of gastric cancer and the survival quality of patients. At present, clinicians mainly evaluate the prognosis of patients with gastric cancer according to the 8^th^ edition of the American Joint Committee on Cancer tumor node metastasis (AJCC-TNM) staging system. However, the prognosis of patients with gastric cancer within the same TNM stage is usually different after receiving similar treatment [6]. Therefore, further studies are needed to identify new tumor markers with high specificity and sensitivity in gastric cancer and to distinguish patient subgroups with a high risk of recurrence and metastasis to accurately predict the prognosis of patients with gastric cancer and determine the optimal therapeutic strategy.

Tumor-related inflammation plays an important role in the occurrence and development of tumors, and immune and inflammatory cells are considered important components in the tumor microenvironment [7]. Immune and inflammatory cells in peripheral blood, such as neutrophils, monocytes, platelets, and lymphocytes, are believed to lead to invasion and metastasis of tumor cells, which have close correlations with the progression of a variety of tumors [8–10]. Some indexes of the above inflammatory cells, such as the neutrophil-lymphocyte ratio (NLR), platelet-lymphocyte ratio (PLR), and monocyte-lymphocyte ratio (MLR), have become prognostic factors for various cancers and are used to predict survival and recurrence of cancers, including gastric cancer [11–14]. Recently, the systemic immune-inflammation index (SII), based on peripheral lymphocytes, neutrophils, and platelet count, has been used to better reflect the balance between host inflammation and immune status; its prognostic value in hepatocellular carcinoma [15, 16], oesophageal cancer [17], colorectal cancer [18], and small cell lung cancer [19] has been confirmed, but its value in gastric cancer remains unclear. In this study, the prognostic value of SII for gastric cancer patients who underwent radical surgery was evaluated, and the prognostic nomogram of resectable gastric cancer was plotted and compared with the traditional AJCC-TNM staging system to determine whether the model can evaluate the prognosis more accurately, since the nomogram has been confirmed in other validation cohorts.

## 2. Materials and Methods

### 2.1. Clinical Data of Patients

A total of 688 patients with gastric cancer receiving radical resection in Fudan University Cancer Hospital from 2012 to 2014 were collected as the primary cohort. Another 174 patients with gastric cancer receiving radical resection in the Third People's Hospital of Yancheng were enrolled as the validation cohort. In this study, all patients had gastric adenocarcinoma, and others with malignant gastric tumors, such as lymphoma, gastrointestinal stromal tumors, and gastric stump carcinoma, were excluded from this study. No patients underwent neoadjuvant chemotherapy. Moreover, patients with active infection or inflammatory diseases within 1 month before blood examination were also excluded. Patients pathologically diagnosed with T3, T4, or lymph node metastasis and high-risk factors (poorly differentiated tumors, lymphatic vascular infiltration, etc.) were treated with fluorouracil-based adjuvant chemotherapy after operation. OS was determined by the period from the time of surgery to the last follow-up or date of patient death. In the primary cohort, the median follow-up time was 36 months (1–75 months), and the number of events for OS was 383 at the last follow-up. In the validation cohort, the median follow-up time was 32 months (4–69 months), and the number of events for OS was 86 at the last follow-up. All patients signed the informed consent, all studies were performed according to the *Helsinki Declaration*, and this retrospective experiment was approved by the Ethics Committee of the Third People's Hospital of Yancheng.

### 2.2. Systemic Inflammatory Indexes

Peripheral blood was collected within 1 week before the operation to detect neutrophils, lymphocytes, and platelet count. Blood cell counts were detected by Sysmex XT-1800i Automated Hematology System (Shanghai, China): NLR = neutrophil count/lymphocyte count; PLR = platelet count/lymphocyte count; MLR = monocyte count/lymphocyte count; SII = platelet count × neutrophil count/lymphocyte count. The optimal cut-off values for the above indexes were obtained using X-tile software (https://x-tile.software.informer.com/) [20]: SII (SII ≤ 320, SII > 320), NLR (NLR ≤ 1.3, NLR > 1.3), PLR (PLR ≤ 135, PLR > 135), and MLR (MLR ≤ 0.23, NLR > 0.23).

### 2.3. Statistical Analysis

SPSS 22.0 software, GraphPad Prism 5.0, and R language were used for statistical analysis. A time-dependent ROC curve was carried out using R software version 3.2.0 (http://www.r-project.org/) with rms and survival ROC packages. Analysis of variance and the Pearson chi-square test were used to evaluate the correlations among variables. The survival curve was plotted using the Kaplan-Meier method, and differences between groups were compared via the log-rank test. Univariate and multivariate Cox proportional hazard models were adopted to determine prognostic indexes, and the nomogram was plotted based on multivariate analysis results. Moreover, the efficiency of the nomogram was evaluated, and the time-dependent receiver operating characteristics (ROC) curve and C index were analysed to compare the discriminatory ability of different models in total survival. Unless otherwise specified, *P* < 0.05 suggested that the difference was statistically significant.

## 3. Results

### 3.1. Clinicopathological Features of the Patient

In the primary cohort, there were 471 males and 217 females, aged 56 years on average, including 316 cases of tumor diameter ≤ 5 cm and 372 cases of tumor diameter > 5 cm. There were 367 cases of high-moderate differentiation, and 321 cases of poor differentiation or no differentiation. In terms of the Lauren type, there were 328 cases of intestinal type, 115 cases of diffuse type, and 245 cases of mixed type. According to the 8th edition of the AJCC-TNM staging system, there were 183 cases in stage I, 215 cases in stage II, and 290 cases in stage III. Four hundred twenty-one patients underwent postoperative adjuvant chemotherapy, while 267 patients underwent no postoperative adjuvant chemotherapy. Other clinicopathological parameters and the validation cohort of patients are shown in Table 1.

The correlation between various systemic inflammatory indexes and clinicopathological features is shown in Table 2. In the primary cohort, the preoperative SII > 320 was related to poor differentiation, upper tumor site, larger tumor, and later TNM stage. In the validation cohort, SII was also related to poor differentiation, larger tumor, and later TNM stage. Moreover, SII had strong correlations with other systemic inflammatory indexes (NLR, PLR, and MLR) in both the primary cohort and the validation cohort (Table 2).

### 3.2. Survival Analysis

**Figure 1:**
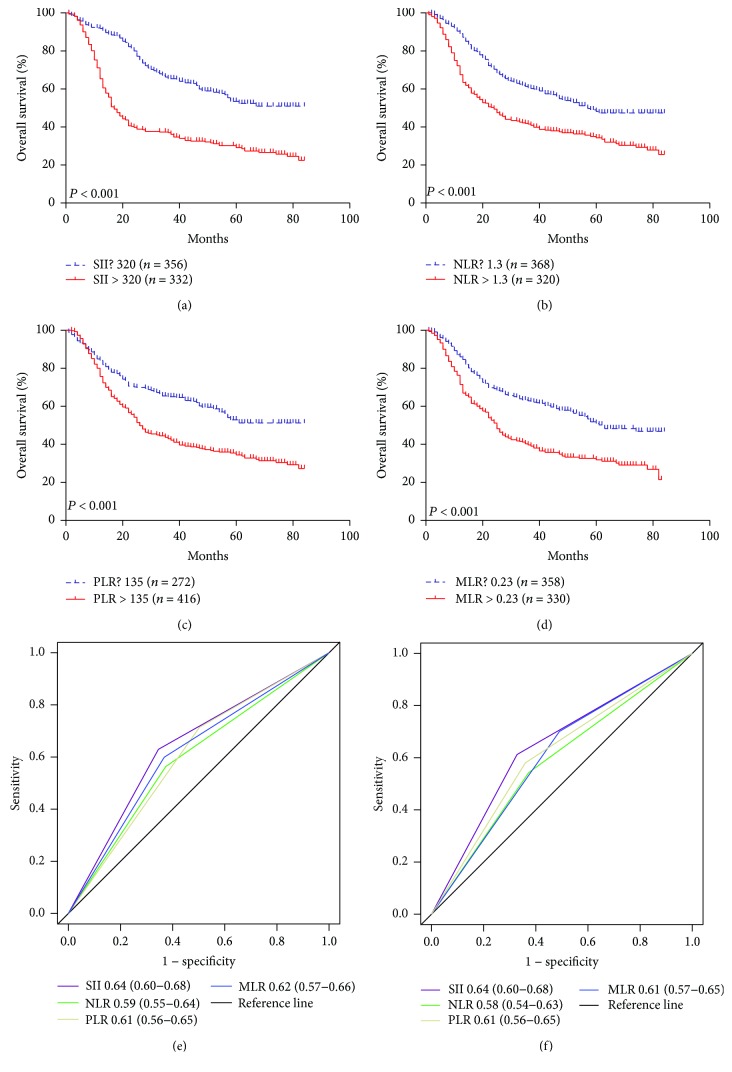
The prognostic significance of the SII (a), NLR (b), PLR (c), and MLR (d) in gastric cancer in the primary cohort. Predictive ability of the SII in gastric cancer was compared with PLR, NLR, and MLR by ROC curves in 3 years (e) and 5 years (f) in the primary cohort.

**Figure 2:**
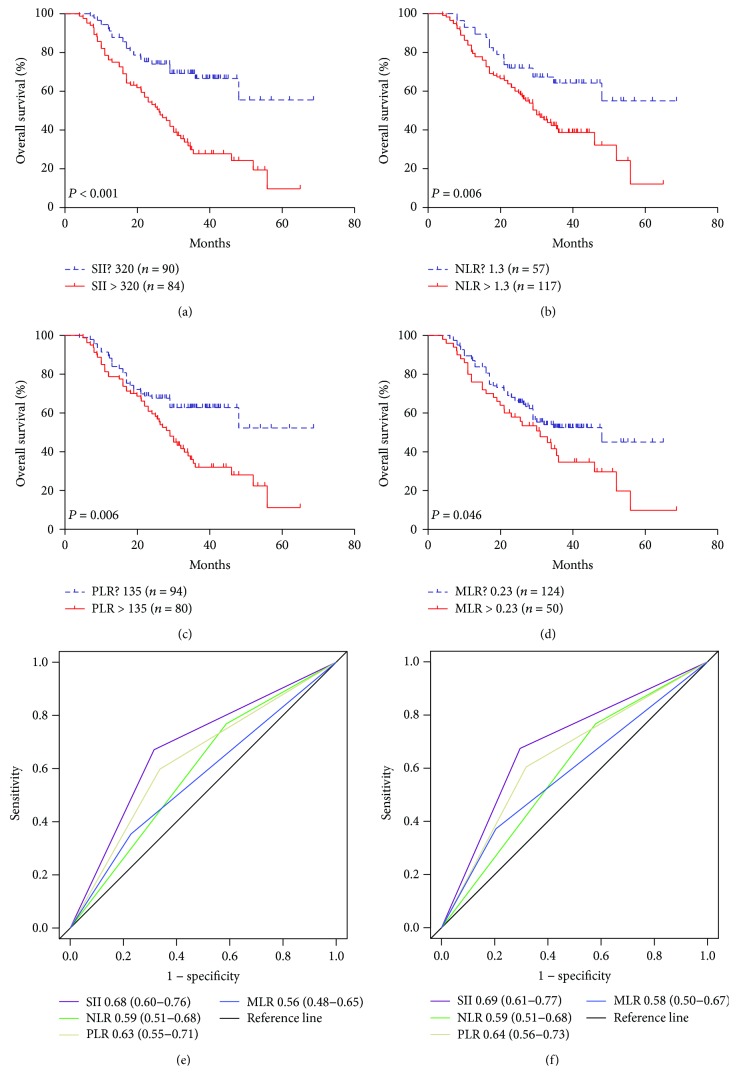
The prognostic significance of the SII (a), NLR (b), PLR (c), and MLR (d) in gastric cancer in the validation cohort. Predictive ability of the SII in gastric cancer was compared with PLR, NLR, and MLR by ROC curves in 3 years (e) and 5 years (f) in the validation cohort.

In the primary cohort, the Kaplan-Meier survival analysis revealed that gastric cancer patients with high SII, PLR, NLR, and MLR scores had poor prognosis (Figures 1(a)–1(d)), but the counts for neutrophils, lymphocytes, platelets, and monocyte alone showed no significant influences on prognosis. The relationships between survival and tumor site, histological grade, tumor size, nerve infiltration, and TNM staging were determined according to the Cox univariate analyses. SII, PLR, NLR, and MLR were factors affecting prognosis, while gender, age, Lauren type, vascular infiltration, and postoperative adjuvant chemotherapy had no significant influences on prognosis (Table 3). Multivariate analysis further revealed that TNM stage and SII were independent risk factors for gastric cancer (Table 3). Among SII, NLR, PLR, and MLR, only SII was an independent risk factor for OS (HR = 1.61, 95% CI: 1.27–2.05, *P* = 0.041). In addition, the ROC curve showed that the area under the curve of SII was larger than that of NLR, PLR, and MLR, indicating that SII is superior to other inflammatory indexes in predicting the 3- and 5-year survival rates of patients with gastric cancer (Figures 1(e)–1(f)). Survival analysis of the validation cohort showed that patients with SII ≤ 320 had longer OS, and similar results were obtained in NLR, PLR, and MLR (Figures 2(a)–2(d)). Among SII, NLR, PLR, and MLR, however, only SII was an independent prognostic index for patients with gastric cancer in the multivariate analysis (HR = 2.94, 95% CI: 1.83–4.17, *P* < 0.001, Table 3). The ROC curve showed that the area under the curve of SII was larger than that of NLR, PLR, and MLR (Figures 2(e)–2(f)). To sum up, it is believed that SII is superior to other inflammatory indexes in predicting the 3- and 5-year survival rates of patients with gastric cancer.

### 3.3. Establishment and Verification of the Nomogram

**Figure 3:**
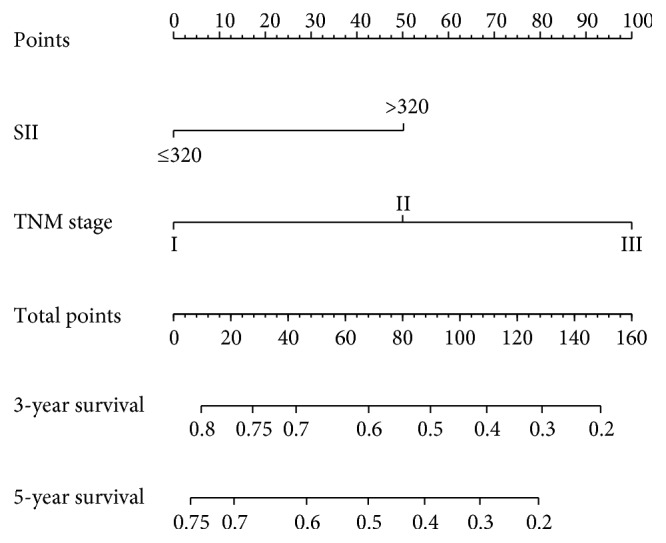
Evaluation of nomogram-integrated SII and TNM stage in patients with gastric cancer.

**Figure 4:**
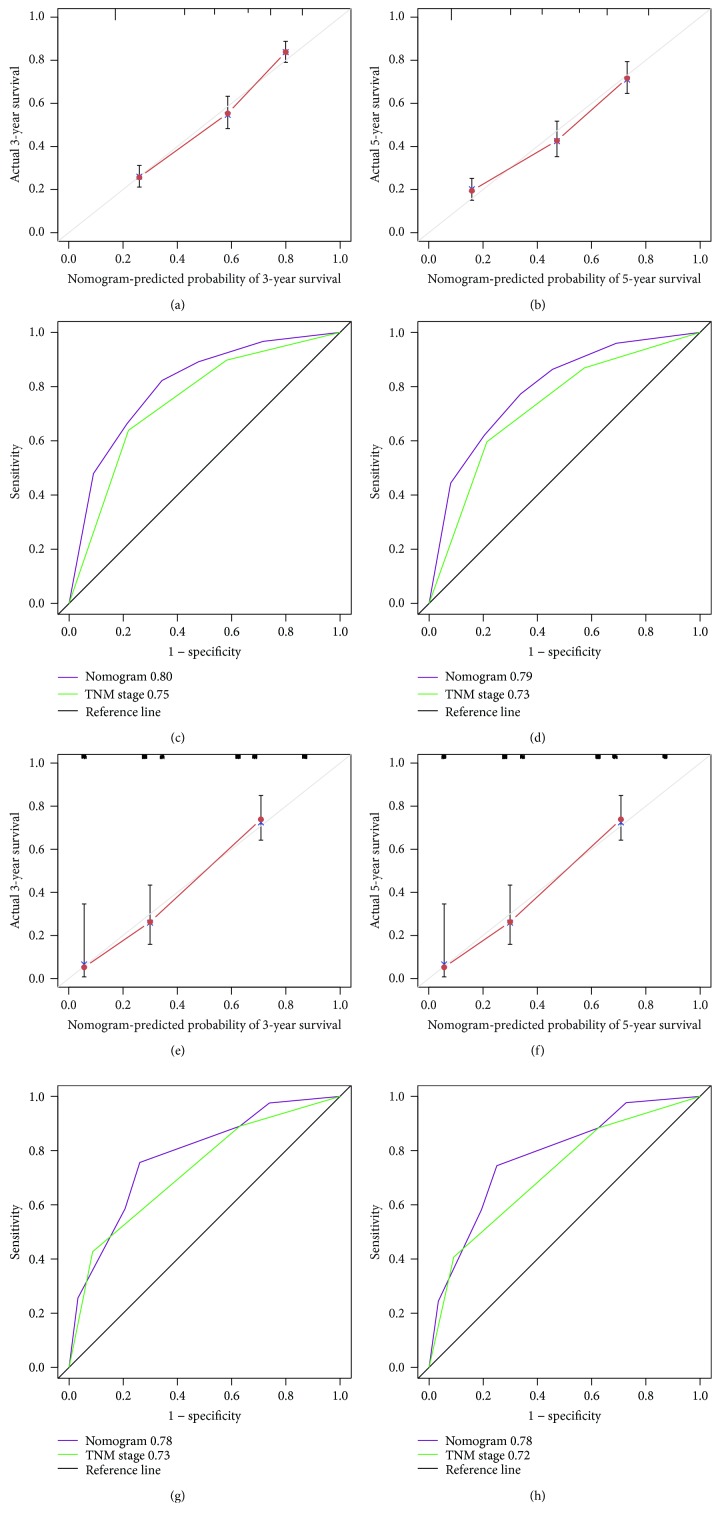
The calibration curve for predicting patient survival at 3 years (a) and 5 years (b) in the primary cohort. Time-dependent ROC curves by nomogram and TNM staging for 3-year (c) and 5-year (d) OS in the validation cohort. The calibration curve for predicting patient survival at 3 years (e) and 5 years (f) in the primary cohort. Time-dependent ROC curves by nomogram and TNM staging for 3-year (g) and 5-year (h) OS in the validation cohort.

Based on the multivariate analysis results, the following variables were eventually integrated in the nomogram to predict the 3- and 5-year survival of the primary cohort: TNM stage and SII (Figure 3). The C index of nomogram was 0.74, which was significantly higher than that of TNM staging 0.70 (*P* < 0.001). The 3- and 5-year survival probability calibration charts showed that the predictive height of the nomogram was consistent with the actual observations (Figures 4(a) and 4(b)). Moreover, the nomogram predicted the survival of the primary cohort more accurately. The area under the ROC curve of the nomogram was significantly larger than that of TNM stage (Figures 4(c) and 4(d)) (*P* < 0.001).

Finally, the established nomogram was used to verify survival in the validation cohort. The calibration curve showed that the 3- and 5-year survival rates predicted by the nomogram were consistent with the actual observation (Figures 4(e) and 4(f)). The C index of the nomogram was 0.72, which was significantly higher than that of TNM staging 0.69 (*P* < 0.001). The area under the ROC curve of the nomogram in the predictive validation cohort was also significantly larger than that of the TNM stage (Figures 4(g) and 4(h)) (*P* < 0.001). The above results indicate that the established nomogram, based on SII, is superior to the TNM staging system in predicting the survival of patients with gastric cancer.

## 4. Discussion

In the 19^th^ century, Rudolf Vichow et al., German pathologists, found leukocytes in tumor tissues and proposed that there was a close correlation between inflammation and tumors [21]. With continuing research, the important correlation between inflammation and tumors has been gradually confirmed by related epidemiological and molecular biology research. According to epidemiological surveys, major clinical evidence for the connection between chronic inflammation and tumors exists. Molecular biological studies distinguish the interaction between inflammation and tumors from phenomenon and mechanisms. Tumor-related inflammatory factors include not only inflammatory factors produced by tumor cells but also tumor-related inflammatory cells and inflammatory factors released during tissue engineering, repair, and angiogenesis [10]. Under the status of injury or infection, the local immune system activates a large number of inflammatory cells, such as macrophages, neutrophils, mast cells, and lymphocytes. These inflammatory cells secrete a variety of cytokines, such as tumor necrosis factor (TNF), IL-6, vascular endothelial growth factor, fibroblast growth factor, platelet-derived growth factor, and extracellular matrix proteins, such as matrix metalloproteinase (MMP), elastase, neutral protease, and collagenase, forming the inflammatory microenvironment and repairing damaged tissues. However, when there is such an inflammatory microenvironment in tumor patients, a large number of inflammatory mediators that can change the stable internal environment will be released, leading to an inflammation-related cascade, tissue atrophy and destruction, and promoting malignant progression of tumors [22, 23]. The persistent inflammatory microenvironment induces tumorigenesis, and the formation and development of tumors further aggravate the inflammatory response. Therefore, systemic inflammatory response has a clear correlation with tumor prognosis, and the prognostic scoring system based on systemic inflammatory response can effectively evaluate the prognosis of cancer patients. NLR, PLR MLR, GPS, and mGPS have been proved to be effective systemic inflammatory score indexes.

In this study, the prognostic value of SII in patients with gastric cancer was studied. SII is a systemic inflammatory index based on neutrophils, platelets, and lymphocytes. Neutrophils are mainly involved in nonspecific cellular immunity of the blood, which can release a large amount of nitric oxide, arginase, and reactive oxygen species (ROS), leading to disorders of T cell activation [24]. Circulating neutrophils have a prognostic value in a variety of tumors, and studies confirm that patients with larger peripheral neutrophilic granulocyte count, or NLR, have a lower survival rate [25, 26]. Platelets can protect the CTC from shear stress, induce epithelial-mesenchymal transition, and promote the overflow of tumor cells to the metastatic site [8, 27]. At the same time, it has been reported that platelets and neutrophils promote adhesion and spread in distant organs through secretion of vascular endothelial growth factor [9, 28]. Lymphocytes are an important component of leukocytes produced in lymphoid organs, which are an important cellular component involved in the immune response. Lymphatic reflux in the body can make the lymph nodes receive a timely antigenic stimulation, secrete a large number of cells and humoural factors, generate specific immune response, and control tumor growth [29]. A decline in the number and function of lymphocytes will weaken immune surveillance and defence from cancer [29].

In this study, SII proved to be related to tumor location, histological grade, tumor size, TNM stage, and perineural infiltration in patients with gastric cancer and was an independent prognostic factor for patients with gastric cancer. SII has a better predictive ability than other existing prognostic indexes based on inflammation, such as NLR, PLR, and MLR. The nomogram established can accurately predict the 3- and 5-year survival rates of patients with gastric cancer after operation, and its accuracy is significantly higher than that of the 8^th^ edition AJCC staging system. There were some limitations in this study, such as selection bias and the retrospective study design. The systemic inflammatory response in the peripheral blood was not compared with local inflammation of tumors. However, the potent prognostic efficacy and convenient detection method of SII as a new systemic inflammatory score enable it to be beneficial. Many previous studies researched that systemic immune indexes can be used to predict efficacy of therapies in tumor patients [30–33], but we have only researched the relationship between the systemic immune indexes and gastric cancer patients' OS. We suggested further research on the relationship between systemic immune indexes and tumor relapse. In addition, the level of these indices in relapse cases and their dynamic changes with treatment response also need to be explored. In the future, a multicentre, prospective study is needed to verify our findings.

In conclusion, this study indicates that as a novel prognostic score based on inflammation, SII can independently predict the overall survival of patients with gastric cancer after operation, which is superior to the existing systemic inflammatory indexes (NLR, PLR, and MLR) that are based on peripheral blood immunity and inflammatory cells. The prognostic nomogram based on SII is a reliable model for predicting the postoperative survival of patients with gastric cancer.

## Figures and Tables

**Table 1:** Clinicopathological characteristics of patients with gastric cancer in the primary cohort and the validation cohort.

Characteristic	Primary cohort (*n* = 688)		Validation cohort (*n* = 174)	
	No. of patients	%	No. of patients	%
Sex				
Male	471	68.5	131	75.3
Female	217	31.5	43	24.7
Age				
≤60	463	67.3	103	59.2
>60	225	32.7	71	40.8
Tumor location				
Upper	106	15.4	28	16.1
Middle	267	38.8	53	30.5
Lower	315	45.8	93	53.4
Histological grade				
Well or moderately differentiated	367	53.3	100	57.5
Poorly or not differentiated	321	46.7	74	42.5
Lauren type				
Diffuse	115	16.7	34	19.5
Intestinal	328	47.7	75	43.1
Mixed	245	35.6	65	37.4
Tumor size				
≤5	316	45.9	72	41.4
>5	372	54.1	102	58.6
Lymphovascular invasion				
No	483	70.2	111	63.8
Yes	205	29.8	63	36.2
Perineural invasion				
No	392	57.0	122	29.9
Yes	296	43.0	52	70.1
TNM stage (AJCC, 8th)				
I	183	26.6	43	24.7
II	215	31.3	88	50.6
III	290	42.2	43	24.7
Adjuvant chemotherapy				
No	267	38.8	62	35.6
Yes	421	61.2	112	64.4

**Table 2:** Baseline characteristics for patients with SII ≤ 320 versus SII > 320 in primary and validation cohorts.

Clinical parameter	Primary cohort				Validation cohort			
	SII ≤ 320 (356)	SII > 320 (332)	*χ* ^2^	*P*	SII ≤ 320 (90)	SII > 320 (84)	*χ* ^2^	*P*
Sex			0.75	0.386			0.07	0.79
Male	249	222			67	64		
Female	107	110			23	20		
Age			0.55	0.457			0.01	0.932
≤60	235	228			53	50		
>60	121	104			37	34		
Tumor location			16.51	<0.001^∗^			1.35	0.51
Upper	36	70			17	11		
Middle	142	125			25	28		
Lower	178	137			48	45		
Histological grade			31.85	<0.001^∗^			8.10	0.004^∗^
Well or moderately differentiated	153	214			61	39		
Poorly or not differentiated	203	118			29	45		
Lauren type			2.15	0.341			3.05	0.218
Diffuse	65	50			17	17		
Intestinal	161	167			34	41		
Mixed	130	115			39	26		
Tumor size			55.11	<0.001^∗^			13.50	<0.001^∗^
≤5	212	104			45	27		
>5	144	228			35	67		
Lymphovascular invasion			1.39	0.238			0.02	0.896
No	257	226			57	54		
Yes	99	106			33	30		
Perineural invasion			6.20	0.013^∗^			0.09	0.766
No	219	173			64	58		
Yes	137	159			26	26		
TNM stage (AJCC, 8th)			83.59	<0.001^∗^			13.99	0.001^∗^
I	119	64			26	17		
II	146	69			35	53		
III	91	199			9	34		
Adjuvant chemotherapy			0.84	0.36			1.55	0.213
No	144	123			36	26		
Yes	212	209			54	58		
NLR			140.83	<0.001^∗^			48.38	<0.001^∗^
NLR ≤ 1.3	268	100			51	6		
NLR > 1.3	88	232			39	78		
PLR			123.64	<0.001^∗^			103.24	<0.001^∗^
PLR ≤ 135	212	60			82	12		
PLR > 135	144	272			8	72		
MLR			38.74	<0.001^∗^			21.60	<0.001^∗^
MLR ≤ 0.23	226	132			78	46		
MLR > 0.23	130	200			12	38		

**Table 3:** Univariate and multivariate Cox regression analyses for overall survival in patients with gastric cancer.

Variables	Univariate analysis		Multivariate analysis	
	HR (95% CI)	*P* value	HR (95% CI)	*P* value
Primary cohort				
Sex: male vs. female	1.14 (0.92–1.41)	0.223		
Age: >60 vs. ≤60	1.24 (0.99–1.54)	0.053		
Tumor location		0.001^∗^		0.057
Middle vs. upper	0.71 (0.54–0.93)	0.018^∗^	0.84 (0.63–1.12)	0.227
Lower vs. upper	0.58 (0.44–0.76)	<0.001^∗^	0.71 (0.53–0.95)	0.020^∗^
Grade: poorly vs. well	0.56 (0.40–0.72)	<0.001^∗^	1.12 (0.89–1.41)	0.350
Lauren type		0.137		
Intestinal vs. diffuse	1.21 (0.91–1.62)	0.195		
Mixed vs. diffuse	0.98 (0.72–1.34)	0.907		
Tumor size: >5 vs. ≤5	2.38 (1.92–2.94)	<0.001^∗^	1.19 (0.94–1.51)	0.142
Lymphovascular: yes vs. no	1.21 (0.97–1.50)	0.086		
Perineural: yes vs. no	1.58 (1.29–1.93)	<0.001^∗^	1.21 (0.96–1.53)	0.101
TNM stage		<0.001^∗^		<0.001^∗^
II vs. I	2.03 (1.46–2.83)	<0.001^∗^	1.77 (1.26–2.47)	0.001^∗^
III vs. I	4.68 (3.46–6.34)	<0.001^∗^	3.10 (2.27–4.24)	<0.001^∗^
Chemotherapy: Yes vs. No	1.12 (0.91–1.37)	0.282		
SII: >320 vs. ≤320	2.47 (2.01–3.04)	<0.001^∗^	1.61 (1.27–2.05)	0.041^∗^
NLR: >1.3 vs. ≤1.3	1.79 (1.47–2.20)	<0.001^∗^	1.25 (0.99–1.58)	0.054
PLR: >135 vs. ≤135	1.82 (1.46–2.27)	<0.001^∗^	1.04 (0.81–1.33)	0.787
MLR: >0.23 vs. ≤0.23	1.88 (1.54–2.31)	<0.001^∗^	1.17 (0.93–1.47)	0.183
Validation cohort				
Sex: male vs. female	1.01 (0.62–1.63)	0.987		
Age: >60 vs. ≤60	1.09 (0.88–1.36)	0.442		
Tumor location		0.046^∗^		0.098
Middle vs. upper	0.51 (0.29–0.89)	0.018^∗^	0.61 (0.30–1.41)	0.146
Lower vs. upper	0.51 (0.27–0.94)	0.032^∗^	0.65 (0.38–1.10)	0.087
Grade: poorly vs. well	1.25 (1.01–1.54)	0.041^∗^	1.48 (0.95–2.29)	0.080
Lauren type		0.386		
Intestinal vs. diffuse	0.65 (0.34–1.25)	0.196		
Mixed vs. diffuse	0.99 (0.63–1.57)	0.979		
Tumor size: >5 vs. ≤5	1.72 (1.09–2.70)	0.019^∗^	1.81 (0.81–4.04)	0.151
Lymphovascular: yes vs. no	1.48 (0.90–2.43)	0.118		
Perineural: yes vs. no	1.70 (1.06–2.72)	0.028^∗^	1.43 (0.61–3.39)	0.414
TNM stage		<0.001^∗^		<0.001^∗^
II vs. I	2.91 (1.45–5.83)	0.003^∗^	3.19 (1.43–3.63)	0.002^∗^
III vs. I	7.12 (3.50–14.50)	<0.001^∗^	7.05 (3.15–15.81)	<0.001^∗^
Chemotherapy: yes vs. no	1.26 (0.79–2.01)	0.001^∗^		
SII: >320 vs. ≤320	2.75 (1.75–4.32)	<0.001^∗^	2.94 (1.83–4.73)	<0.001^∗^
NLR: >1.3 vs. ≤1.3	2.03 (1.23–3.35)	0.006^∗^	1.71 (0.96–3.39)	0.067
PLR: >135 vs. ≤135	1.90 (1.24–2.94)	0.004^∗^	1.46 (0.93–2.30)	0.102
MLR: >0.23 vs. ≤0.23	1.56 (1.02–2.40)	0.046^∗^	1.22 (0.76–1.95)	0.420

## Data Availability

The data used to support the findings of this study are available from the corresponding author upon request.
